# A systematic review of the association between history of sexually transmitted infections and subsequent condom use in adolescents

**DOI:** 10.1186/s12889-024-18322-2

**Published:** 2024-04-10

**Authors:** Frédérique Tremblay, Yohann Courtemanche, Richard E. Bélanger, Anne-Marie Turcotte-Tremblay

**Affiliations:** 1 Department of Social and Preventive Medicine, Faculty of Medicine, Université Laval, 1050, avenue de la Médecine, Room 4633, Québec, (QC) G1V 0A6 Canada; 2 Projet COMPASS Québec, VITAM – Centre de recherche en santé durable, CIUSSS-CN and Université Laval, GMF-U Maizerets, 2480, chemin de la Canardière, Québec, (QC) G1J 2G1 Canada; 3 Faculy of Nursing, Université Laval, 1050, avenue de la Médecine, Room 3645, Québec, (QC) G1V 0A6 Canada; 4 Department of Pediatrics, Faculty of Medicine, Université Laval, 1050, avenue de la Médecine, Room 4633, Québec, (QC) G1V 0A6 Canada

**Keywords:** Adolescents, Sexually transmitted infections, Condoms, Preventive behaviors, Sexual behaviors, Health Belief Model, Systematic review, Epidemiology

## Abstract

Sexually transmitted infections (STIs) are common among adolescents. According to the Health Belief Model, cues to action influence preventive behaviors. Cues to action can include health experiences such as being diagnosed with an STI. The impact of a history of STIs on subsequent condom use among adolescents remains largely unexamined, despite high rates of recurrence and their health impacts. This project aimed to systematically review the literature on the association between curable STIs and subsequent condom use among adolescents. The systematic review, reported following PRISMA guidelines, was conducted using the Joanna Briggs Institute method. Eligible studies, in the form of cohort studies, case-control studies, or cross-sectional studies, targeted adolescents aged 10 to 24, with or without a history of curable STIs; the outcome was subsequent condom use. MEDLINE (Ovid), Embase (Elsevier), and Web of Science were searched from January 2012 to December 2022 with the assistance of an information specialist. Two reviewers independently selected articles and extracted data. Risk of bias analysis was performed using ROBINS-E. The review explores results, with tables, based on population characteristics, exposure, and outcome, and addresses the influence of gender, ethnicity, and age. Of 3088 articles identified, seven studies were retained. Almost all the studies focused on African-American, Nigerian, or Rwandan adolescents, and several included only girls. Among girls, a history of STI increased subsequent condom use in combination with other contraceptive methods (*n* = 4). Among boys and older adolescents of both genders, a history of STI was associated with a decrease in condom use (*n* = 3). No study distinguished between different STIs. While all the studies (*n* = 7) presented a high risk of bias, six did not present a threat to conclusion validity. All the studies indicated that a history of STI could influence subsequent protective behaviors, possibly by acting as a cue to action, as posited by the Health Belief Model. This information enhances our understanding of factors leading to the adoption of preventive health measures among adolescents and could apply to other infectious experiences.

**Registration** The protocol is registered in PROSPERO (CRD42023397443).

**Supplementary Information:**

The online version contains supplementary material available at 10.1186/s12889-024-18322-2.

## Background

Sexually active adolescents do not always use condoms during sex. Indeed, while more than 50% of 15–24-year-olds have experienced their first sexual intercourse by age 18 [1], 40–50% of youths nevertheless report not having used condoms during their most recent sexual intercourse [2, 3], which increases their risks of acquiring STIs. Worldwide, there are approximately 333 million new cases of STIs annually, with the highest rates occurring among 15–24-year-olds [4]. STI incidence is rising, with the largest increase being in adolescents [5, 6]. Adolescents represent at least one-third of cases of chlamydia, with the highest levels being seen in younger adolescent girls [4]. Adolescents and youths between the ages of 10 and 24 years [7] are particularly susceptible to STIs due to biological, behavioral, and social factors [8, 9]. Adolescents from lower socioeconomic backgrounds [10], ethnic minorities [11], and sexual orientation minorities [12] face even higher odds of infection.

Condoms, used during sexual intercourse, are effective in reducing STIs by acting as a barrier to transmission [13, 14]. Some STIs remain asymptomatic but transmissible [15]. Transmission can occur through vaginal, anal, or oral sex [15]. Chlamydia (including lymphogranuloma venereum, or LGV), gonorrhea, syphilis, and trichomoniasis are curable [16], and condoms remain the most effective method for reducing the risks of those STIs during sex [17].

Not using condoms during sex can lead to STIs, which, if left untreated, may result in severe physical consequences such as blindness, cancer, cardiovascular diseases, sterility, and even death [18]. While adolescents may not frequently experience these consequences, they may develop such problems later in adulthood [18]. Worldwide, 50% of the most common STIs are curable (chlamydia, gonorrhea, syphilis, and trichomoniasis), with the most common being chlamydia [19]. Gonorrhea is the second most reported bacterial STI [19] and, although rare, can cause infertility in both sexes if untreated [20]. Clinical conditions can include pelvic inflammatory disease, chronic pelvic pain, ectopic pregnancy in girls, and epididymitis in boys [20]. STIs also entail an important economic burden. For example, the 26 million new STIs in 2018 in the United States of America (USA) are estimated to have incurred $16 billion in direct medical costs for the American healthcare system [21, 22], with 15–24-year-olds accounting for up to 26% of the total cost [22]. Therefore, understanding the factors that influence preventive health behaviors in this area has important implications for the healthcare system.

In public health, the Health Belief Model (HBM) is often used to explain health-related behaviors [23, 24], such as condom use. In the HBM, the adoption of preventive health behaviors is influenced indirectly by cues to action, which can be events experienced by the individual [23, 24]. According to that model, a history of STI could influence an individual’s subsequent protective behavior by acting as a cue to action such as using a condom during sexual intercourse. Studies in behavioral change psychology and in neurobiology suggest that memories of a past STI could activate or alter subsequent actions, such as the adoption of protective sexual behavior [25, 26].

Understanding the impact of an STI on subsequent condom use in adolescents could be particularly important. In one study, a history of STI and related shame were positively associated with condom use in African-American female adolescents [27]. However, in another, condom use was negatively associated with history of STI in sexually active adolescents [28]. In a 2002 survey in the USA, adolescents with a history of STI reported less condom use at most recent intercourse [29]. Other studies on this association have reached conflicting conclusions, with a history of STI associated with either higher or lower subsequent use of condoms [30–36]. Adolescents who have had curable infections may use condoms differently from those with chronic infections, as the latters’ motivation may also be linked to the protection of uninfected partners [37]. Furthermore, even curable infections can recur, which can be avoided if adequate behavior changes are implemented [38]. Consequently, a synthesis and critical appraisal of the scientific literature on the relation between a history of curable STIs and subsequent condom use in adolescents would be useful to clarify the state of knowledge.

To the best of our knowledge, there is no systematic literature review that would provide a complete picture of the influence of curable STIs on condom use in adolescents. A better understanding of the impacts of those STIs on subsequent condom use would help guide clinical and public health approaches to effective interventions in this population particularly susceptible to STIs and reinfections [39]. Our review will focus on those infections as cues to action related to condom use in adolescents. It will provide information on factors influencing their sexual preventive behaviors that could be of interest to professionals seeking to help adolescents improve their responses to STIs that could have serious consequences for their future health. Hence, the research question is: what effect does a history of STI have on subsequent condom use in adolescents? In particular, the objective of this review is to synthesize and critically appraise the literature on the association between curable STIs and subsequent condom use among adolescents.

## Methods

The protocol for this review is registered in PROSPERO (CRD42023397443) [40]. The Preferred Reporting Items for Systematic reviews and Meta-Analyses (PRISMA) guidelines were followed [41]. The Joanna Briggs Institute (JBI) method for a systematic review of etiology was used [42].

### Eligibility criteria

The eligibility criteria for the studies refer to the JBI method [42], which is the PECOSS approach (P = population, E = exposure C = comparison (non-exposure), O = outcome, S = study design, S = study setting).

#### Population

Adolescents were the target study population. The age range used was that defined recently by Sawyer et al. in *The Lancet*, i.e., 10–24-year-olds [7]. In our protocol, we had predetermined that if the age range was not reported in the article, the population was considered to be within the age range if it concerned “high school students”, “adolescents”, or “young adults”. However, all included studies reported the ages of their participants. Papers reporting exclusively on younger children and/or adults were excluded. Studies had to report on sexual activity. Sexually active youth were defined as having had at least one type of sexual contact (e.g., vaginal, anal, oral) with another person of any gender [43] as self-reported [44]. Papers only about sex workers (e.g., exotic dancers, adult film performers) were excluded, on the basis that their activities involve significantly more risky sexual behaviors and higher risk of contracting STIs than those of adolescents in the general population [45].

#### Exposure

Eligible STIs included curable infections (chlamydia or LGV, gonorrhea, syphilis, and trichomoniasis), which represent the most frequent infections in the adolescent population [16, 46]. Articles focusing only on incurable STIs (HIV, genital herpes, or HPV) were excluded, as this review was aimed specifically at behaviors that could avoid STI recurrences [38], which could not be studied in chronically infected individuals. Studies that included multiple curable STIs or that did not distinguish between curable and incurable STIs (e.g., “do you have an STI?”) were eligible for this review. Both objective (i.e., laboratory results or diagnostic tests) and subjective (i.e., self-reported) measures for the STI were acceptable for inclusion. History of STI among some participants included in the study was required for eligibility.

#### Comparison (non-exposure)

Non-exposure was considered as adolescents’ not having experienced an STI during their lifetime.

#### Outcome of interest

The outcome of interest was condom use measured after an STI among those who had experienced STIs. Condom use was defined as using or not using a condom during vaginal, anal, or oral sex [47]. Condom use had to be stated as an outcome of interest in the studies. Papers about unprotected intercourse or composite measures including condoms were eligible. Eligible condom types included any type of equally effective condom (male/female condoms) [48–51]. Although any type of condom use reporting was allowed, condom use is most often self-reported [52]. Any type of measurement was included (yes/no, count, proportion, etc.), any recall period, any partner specificity, and any sexual act [17].

#### Study design

Observational studies were required [42, 53]. Studies were included if they reported results specifically on the association of interest (STI history and subsequent condom use). Prospective and retrospective cohort studies, case-control studies, and cross-sectional studies were included in the review [53]. Cross-sectional studies were considered if it was clear that individuals who had experienced an STI reported on their infection history and subsequent condom use.

#### Study setting

To obtain a generational portrait of adolescents, studies published from January 2012 to December 2022 were included in the review. The decision was taken to cover the last decade, as major behavioral trends can differ between generations that are exposed to different historical events and context during particular life stages [54, 55].

### Information sources

MEDLINE (Ovid), Embase (Elsevier), and Web of Science were searched [56, 57]. All the reference lists of the included studies from the databases were evaluated for inclusion. A list of all the included studies (*n* = 7) was forwarded to the systematic review team. Only published studies were searched; given the etiological perspective of the systematic review, the grey literature was not considered.

### Search strategy

The search strategy (see Additional file 1) was developed through an iterative process between FT (first author) and the information specialist and subsequently approved by the systematic review team. It was then performed in the selected databases. The concepts considered were: adolescent population, STIs (gonorrhea, syphilis, chlamydia or LGV, and trichomoniasis), condom use, and observational studies, as indicated in the study design section above. The search strategy for observational studies was inspired by the strategies used by Li et al. [58] and Avau et al. [59]. Cross-sectional studies in the form of surveys or questionnaires were included [58, 59]. Given the evolution in terminology in recent years, the term sexually transmitted disease (STD) was included, along with STI, in the literature search. No restrictions were applied on language and year of publication.

### Selection process

The selection process was completed in four steps using Covidence. The first consisted of a pilot selection from 10% of the total number of unique references, randomly chosen. Two reviewers independently selected studies based on the eligibility criteria. This pilot selection ensured a shared comprehension of the eligibility criteria among all members involved in the selection process, using the kappa statistic (> 0.7 considered satisfactory) [60, 61]. When the conclusion was unsatisfactory, the criteria were adjusted. The second step was the selection by two reviewers once the pilot test was concluded. The two reviewers independently selected studies based on titles and abstracts. When there was consensus, the article was included or excluded [62] for full-text screening. The third step consisted of independent selection based on full-text screening by two reviewers, with disagreements resolved through discussion. If disagreements were due to a difference in interpretation, arbitration by a third reviewer was sought. No contact with the authors of the studies was needed. Agreement was again assessed using the kappa statistic [60]. In the fourth step, the included studies were discussed by the reviewers for final selection.

### Data collection process and data items

To extract the variables of interest, an Excel [63] data extraction form was developed based on a coding guide that included definitions of those variables and the extraction modalities. The following variables were considered, in five categories: 1) *study characteristics* included name of first author, year of publication, country in which the study was conducted, study setting, and study design; 2) *population characteristics* included race/ethnicity, gender, sample size, mean, minimum and maximum age, sexual activity, and socioeconomic status; 3) *STI history characteristics* included type of STI, frequency of STI history, type of measure of STI history, and recall period; 4) *condom use characteristics* included frequency of outcome use (for each possible outcome: condom use alone, dual method, multiple methods, unprotected sex acts, protected sex acts, consistent condom use, and risky sexual behavior), condom type, temporal period for each possible outcome, types of response choices presented for condom measurement questions, consistency of condom use, type of sexual act in which the condom outcome was used, abstinence, type of partner, effect measure used (i.e., odds ratio, relative risk), group in which the effect was assessed, adjusted and crude effect measures, and standard error for further calculation of the 95% confidence interval (CI); and 5) *characteristics of the method used* included the regression model for each outcome and the handling of missing data. Pilot extraction was carried out independently on two studies by two reviewers prior to the main data extraction to ensure a shared comprehension and fine-tuning of the extraction guide as needed. The two reviewers then independently carried out the extraction. Disagreements were discussed and resolved with the help of a third reviewer as needed.

### Study risk of bias assessment

Risk of bias in the studies was independently assessed by two reviewers after a pilot step was concluded. Since only cohort and cross-sectional studies were identified, risk of bias was assessed using the Risk Of Bias In Non-randomized Studies—of Exposure (ROBINS-E) tool [64]. This tool covers seven domains of risk of bias related to: 1) confounding; 2) measurement of exposure; 3) selection of participants into the study or into the analysis; 4) post-exposure interventions; 5) missing data; 6) measurement of the outcome; and 7) selection of the reported result [64]. Each of these domains and overall risk of bias were rated as *low risk of bias*, *some concerns*, *high risk of bias*, or *very high risk of bias*, according to the ROBINS-E algorithms [64]. The conclusion of those algorithms could be overridden if the authors of the review deemed that it did not yield an appropriate risk of bias judgment, as recommended by the ROBINS-E Development Group (2023) [64]. Disagreements were discussed and resolved by the two reviewers, with arbitration by a third reviewer as needed. Confounders were selected based on an a priori literature screening, which identified the following as determinants of exposure and outcome: social and sexual network [65, 66]; risky sexual behavior tendencies [67, 68]; education [18, 69, 70]; knowledge and awareness [71–73]; socioeconomic status [65, 74]; healthcare resources [65, 74, 75]; age [8, 9, 65]; gender [76, 77]; ethnicity [11, 78]; and cultural and religious beliefs [65, 79].

### Data synthesis

The study selection process was reported with the number of identified studies, the number of studies retained based on title and abstract, and the number of studies selected by full-text screening based on the eligibility criteria. The extracted data were synthesized using tables and in narrative form regarding the studies, population, exposure, and outcomes characteristics. The associations between STI history and different types of condom use outcomes were individually explored to assess the impact of STI history on different behaviors in different contexts. Further narrative exploration of the associations was based on different outcomes, gender, race/ethnicity, and age. The effect of an STI infection on condom use by adolescents was reported with the effect measure used in the eligible studies and its 95%CI. Where missing, the 95%CI was calculated [80]. When a study did not present an adjusted effect measure, one was calculated, if possible, when data were available. Authors were contacted if information necessary for analysis was missing in the reviewed papers. Given the high heterogeneity of the studies, no meta-analysis was performed. A table was produced presenting the risk of bias of each study according to the ROBINS-E and the relevant domain. Risk of bias was considered in the interpretation of results. Certainty assessment, publication bias assessment, and subgroup analyses were not conducted.

## Results

### Study selection process and study characteristics

**Fig. 1:**
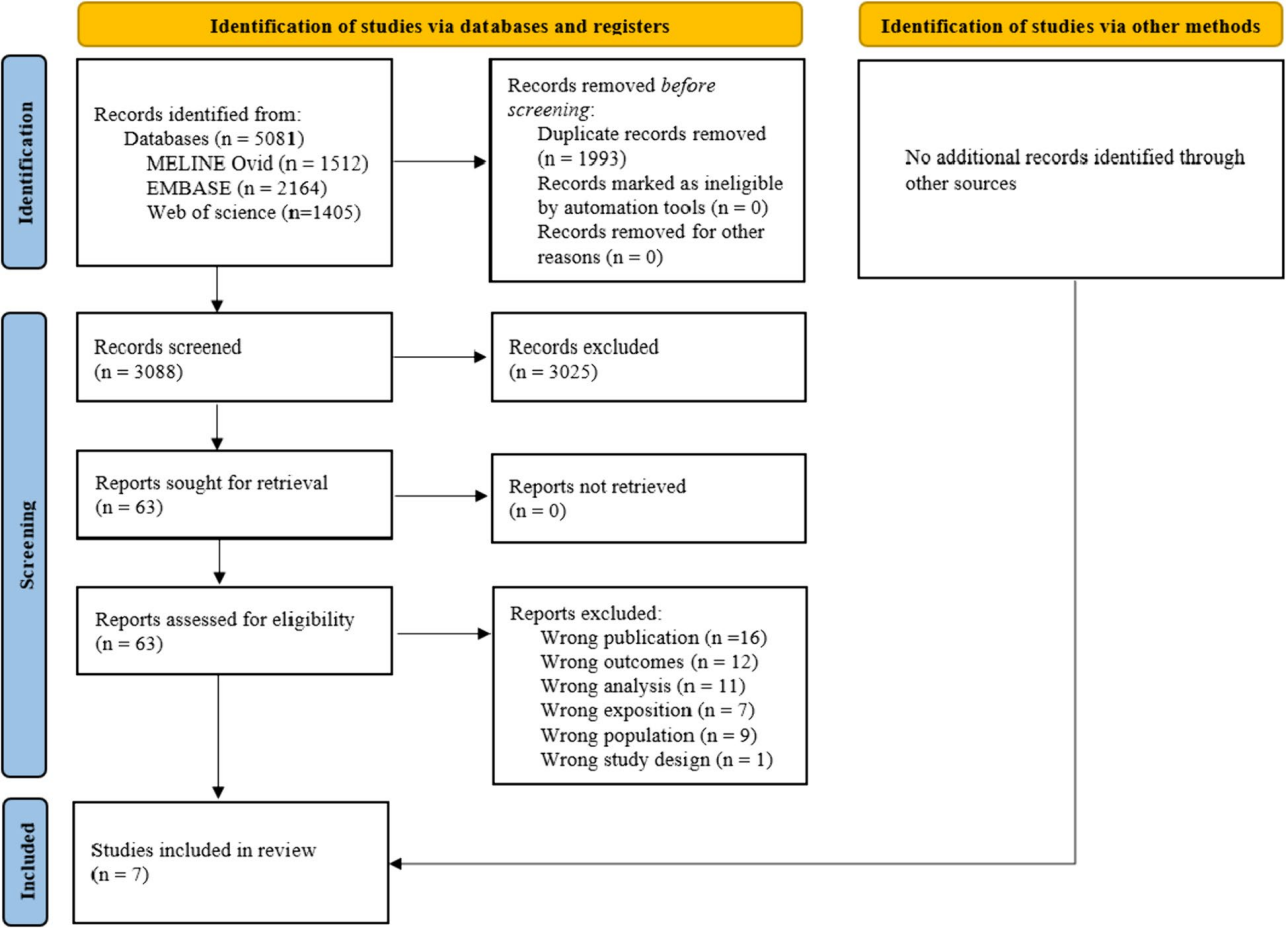
PRISMA 2020 flow diagram for systematic review [41]

From a total of 3088 articles retrieved, seven (Walsh et al. 2014; Kottke et al. 2015; Wallace et al. 2015; Clarke et al. 2016; Chambliss et al. 2021; Ebuenyi et al. 2021; and Kawuki et al. 2022) met the criteria (Fig. 1) [33, 36, 81–85]. Among these included studies, five were cross-sectional [33, 36, 82–84], one was a repeated cross-sectional study [81], and one was a prospective cohort study [85]. The included studies were published between 2014 [85] and 2022 [83], and were conducted in the USA (*n* = 5) [33, 36, 81, 84, 85], in Nigeria (*n* = 1) [82], and in Rwanda (*n* = 1) [83] (Table 1). Three studies were conducted in clinical settings [33, 36, 84], two were community-based, either in an ongoing survey or a demographic survey [81, 83], and two were conducted in schools [82, 85] (Table 1).

**Table 1:**
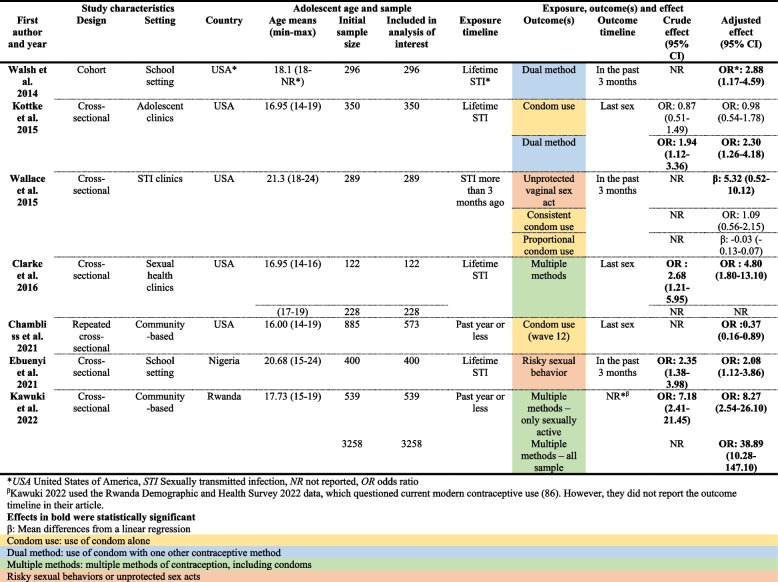
Characteristics of included studies (*n* = 7) [86]

#### Characteristics of the study population

The mean age of participants ranged from 16.0 [81] to 21.3 years [36] (Table 1), with an age range of 14 to 24 years (Table 1). One study [85] did not report the maximum age. All studies reported on origins [33, 81–84] or race [36, 85] (Table 2). Participants were African-American [33, 81, 84], a majority of Black non-Hispanic (61% of the sample) [36], only Nigerian [82], or only Rwandan [83], and in one study, 71% of participants were White [85] (Table 2). Five studies reported on some socioeconomic indicators [33, 82–85], while two reported no information on socioeconomic indicators. The most frequent indicator used was education (*n* = 3) of either a family member [84] or the participant [82, 83]. Gender was reported by all studies, with four studies about girls only [33, 83–85], one about boys only [81], and two about both [36, 82] (Table 2). Six of the seven studies only included sexually active participants [33, 36, 81, 83–85] and limited the analysis to them, while one study included sexually abstinent participants in the main analysis [82] (Table 2).

**Table 2:** Population characteristics of included studies and sexually transmitted infection history (*n* = 7)

**Study** (n^a^)	**Ethnicity or race reporting**	**Ethnicity or race- %**	**Socioeconomic grouping**	**Socieconomic groups—% or mean (SD**^**b**^**)**	**Women (%)**^**c**^	**Sexually active participants (%)**	**Analysis limited to sexually active participants**	**Participants with STI history (%)**^**d**^
**Walsh et al. 2014** (*n* = 296) [85]	Race	White – 71.0	Socioeconomic scale	Mean family socioeconomic status (range 1–10): 6.3 (1.6)	100.0	100.0	Yes	3.0
		Black – 13.0						
		Asian – 8.0						
		Other – 7.0						
**Kottke et al. 2015** (*n* = 350) [84]	Origin	African-American – 100.0	Education	Mother completed high school or GED^b^ – 68.2	100.0	100.0	Yes	43.7
**Wallace et al. 2015** (*n* = 289) [36]	Race	Black non-Hispanic – 61.0	None	-	54.0	100.0	Yes	28.0
		White non-Hispanic – 26.0						
		Other/Multi-race – 8.0						
		Hispanic – 7.0						
**Clarke et al. 2016** (*n* = 350) [33]	Origin	African-American – 100.0	Insurance	Insured (17–19 years-old) – 46.3	100.0	100.0	Yes	43.7
				Insured (14–16 years-old) – 28.9				
				Uninsured (17–19 years old) – 18.3				
				Uninsured (14–16 years old) – 6.0				
**Chambliss et al. 2021** (*n* = 573) [81]	Origin	African-American – 100.0	None	-	0.0	100.0	Yes	NR^b^
**Ebuenyi et al. 2021** (*n* = 400) [82]	Origin	Nigerian – 100.0	Education	Undergraduate – 81.0	59.3	78.0	No	18.0
				Postgraduate – 19.0				
**Kawuki et al. 2022** (*n *= 539) [83]	Origin	East Rwandan – 31.9	Education	Primary – 63.4	100.0	100.0	Yes	2.6
		South Rwandan – 21.3		Secondary – 33.0				
		West Rwandan – 18.6		No education – 3.8				
		North Rwandan or Kigali – 28.0		Tertiary – 0.7				

^a^n = number of youths included in analysis of interest

^b^*SD* Standard deviation, *GED* General education development, *NR* Not reported

^c^All papers included in this review reported results only for two genders (women and men)

^d^No studies differentiated between any type of STI (including incurable STI)

#### Characteristics of STI history

None of the studies differentiated among STI types or noted whether the STI was curable or not [33, 36, 81–85]. Questions were often general, with participants being asked: “Ever been told you had an STD” [36, 81] or whether they had a “previous STD” [84]. Almost all the studies (*n* = 6) reported on what proportion of the participants had a history of STI [33, 36, 82–85], ranging from 2.6% to 43.7% (Table 2). All studies asked about STI history through survey questions [33, 36, 81–85]. Regarding recall, four studies asked about lifetime exposure [33, 82, 84, 85], two asked about history of STI within the past year [81, 83] and one asked specifically about more than three months ago [36] (Table 1).

#### Characteristics of condom use

##### Outcomes

Three types of outcomes regarding condom use were identified: 1) *use of condom alone* (condom use [81, 84], consistent condom use [36], and percentage of protected vaginal sex acts out of the total number of sex acts [proportional condom use] [36]); 2) *use of condom simultaneously with a contraceptive method* (dual method [84, 85], or *multiple methods* of contraception including condoms [33, 83]); and 3) *unprotected sex* (number of unprotected sex acts [36] or risky sexual behaviors [82]) (Table 1). In the three studies reporting on the use of condom alone, the proportion of participants using condoms ranged from 20.6% to 56.9% [81, 84] (Table 3). Only one study reported the proportion of participants consistently using a condom (19.0%); this study also reported 54.7% of proportional condom use [36] (Table 3). Four studies also used dual method or multiple methods as outcomes [33, 83–85]. Of the studies reporting on frequency of use, dual or multiple methods were used by 17.4% to 63.1% of participants [33, 83, 84] (Table 3). Two studies reported on unprotected sex acts, either reporting the number of unprotected vaginal sex acts in the past three months (mean (SD): 12.0 (18.7)) [36] or the proportion of participants using risky sexual behaviors, including condomless sex (46.8%) [82] (Table 3). In the remainder of this paper, “condom use” is an umbrella term for all condom use possible outcomes.

**Table 3:** Characteristics of outcome(s) of included studies (*n* = 7)

**Study** (n^a^)	**Outcome(s)**	**Participants using outcomes % or mean (SD**^**b**^**)**	**Type of condom and sexual act**	**Type of measure**	**Report on consistency**	**Type of partner**	**Analysis**	**Missing values**
**Walsh et al. 2014** (*n* = 296) [85]	Dual method	NR^b^	Undifferentiated – vaginal	Dichotomous (yes/no)	Yes	Other	Multilevel modeling	Multiple imputation
**Kottke et al. 2015** (*n* = 350) [84]	Condom use	20.6	NM^b^—NM	Dichotomous (yes/no)	No	NM	Generalized estimating equations with multinomial outcome	No missing values
	Dual method	20.6						
**Wallace et al. 2015** (*n* = 289) [36]	Number of unprotected vaginal sex	12.0 (18.7)	NM – vaginal	Continuous (number of times condoms were used in a given period)	Yes	NM	Linear regression	No missing values
	Consistent condom use	19.0					Logistic regression	
	Proportional condom use (= 1)	54.7 (35.7)					Linear regression	
**Clarke et al. 2016** (*n* = 350) [33]	Multiple methods	63.1	NM – vaginal	Dichotomous (yes/no)	No	NM	Logistic regression	No missing values
**Chambliss et al. 2021** (*n* = 573) [81]	Condom use (wave 12)	56.9^c^	NM – NM	Dichotomous (yes/no)	No	NM	Logistic regression	Exclusion
**Ebuenyi et al. 2021** (*n* = 400) [82]	Risky sexual behavior	46.8	NM – NM	Dichotomous (yes/no)	No	NM	Logistic regression	No missing values
**Kawuki et al. 2022** (*n* = 539) [83]	Multiple methods	17.4	Male condom – NM	Dichotomous (yes/no)	No	NM	Logistic regression	No missing values

^a^n = number of youths included in analysis of interest

^b^*SD* Standard deviation, *NR* not reported, *NM* not mentioned

^c^This reporting refers to some young people with data who were not included in the final analysis due to missing values. The initial sample for the 12^th^ wave was 885

Condom use: use of condom alone

Dual method: use of condom with one other contraceptive method

Multiple methods: multiple methods of contraception, including condoms

##### Characteristics of reporting

Five studies did not mention condom type [33, 36, 81, 82, 84], one specifically referred to male condoms [83], and one did not differentiate between types of condoms [85] (Table 3). Last sex [33, 81, 84] and sex in the past three months [36, 82, 85] were each used three times for the outcome timeline (Table 1), while one study did not mention any timeline [83]. Almost all studies (*n* = 6) asked about the use of condoms using a dichotomous yes/no question [33, 81–85], while one asked about number of times condoms were used during a given time period [36] (Table 3). Only two studies reported on consistency of condom use [36, 85], and none clearly addressed the type of partner with regard to the sexual activity under study (Table 3). Three studies indicated the type of sex act, all vaginal [33, 36, 85] (Table 3).

#### Characteristics of statistical methods

Five studies used logistic regression [33, 36, 81–83], one used linear regression [36], one used generalized estimating equation to account for dependency in observations [84], and one used multilevel modeling [85] (Table 3). Five studies had no missing values [33, 36, 82–84], one excluded participants based on missing values [81], and one practiced multiple imputation [85] (Table 3).

### Risk of bias

**Fig. 2:**
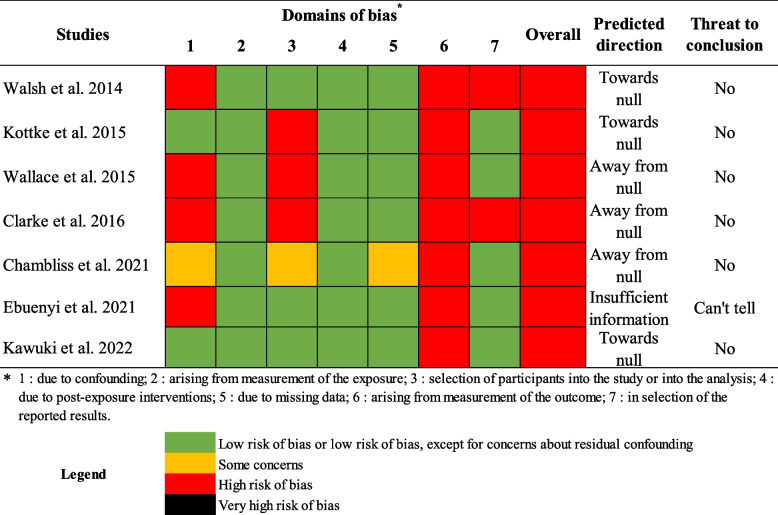
Risk of bias assessment with the Risk of Bias In Non-randomized Studies – of Exposure (ROBINS-E)

Figure 2 presents the risk of bias assessment of the included studies. All studies presented a high risk of bias [33, 36, 81–85]. Five presented some concerns or a high risk of bias due to confounding [33, 36, 81, 82, 85] from a lack of consideration of confounders, which could lead to underestimation of the effect measure. No study presented a risk of bias arising from the measurement of the exposure. One study presented some concerns regarding the selection of participants into the study, while the three studies conducted in a clinical setting (either adolescent clinics or STI clinics) presented a high risk of bias [33, 36, 84]. No bias was found regarding post-exposure intervention, and only one study presented concerns due to missing data [81]. All studies presented a high risk of bias arising from the measurement of condom use, given the possibility of differential information bias regarding outcomes as reported by people with an STI history and those without. Two studies presented a high risk of bias regarding the selection of reported results [33, 85]. Despite the biases identified, most studies still produced interpretable conclusions with respect to predicted directions and the context leading to the conclusions, particularly regarding the 1^st^ and 6^th^ risk of bias domains. Considering this, studies that were classified as having a very high risk of bias using the ROBINS-E’s algorithm were classified as having a high risk of bias. Three studies probably had a larger effect measure than they should have had, while three noted a weaker effect than they should have had.

### Association between history of STI and condom use outcomes

The exposure and outcome timelines and types of outcomes are presented in Table 1, along with crude effects and adjusted effects with 95%CI of the association between an STI history and subsequent condom use, where reported by individual studies. Other details about exposure and outcomes can be found in Tables 2 and 3 respectively.

### Results of synthesis

#### STI history and condom use alone

Three studies examined the association between STI history and condom use alone [36, 81, 84] (Table 1). When condom use alone was observed, only one study found that STI history in the past year was associated with a reduction in condom use at last sex (OR 0.37, 95%CI; 0.16–0.89) [81] (Table 1). This effect was only found in one wave of a repeated cross-sectional study. This study was rated with a high risk of bias without threat to conclusion validity (Fig. 2). Other studies examining the association between STI history and condom use alone did not find a similar association [36, 84] and were also rated with a high risk of bias (Fig. 2).

#### STI history and dual method

Two studies examined the association between STI history and the dual method [84, 85]. When condom use was coupled with another method to prevent pregnancy, one study found statistically significant higher odds of dual method use at last intercourse (adjusted odd ratios [AOR] 2.30, 95%CI; 1.26–4.18) with a lifetime history of STI [84]. The other found higher odds of dual method use in the past three months (AOR 2.88, 95%CI; 1.17–4.59) with a lifetime history of STI [85] (Table 1). While both studies had a high risk of bias, no threat to conclusion validity was found (Fig. 2).

#### STI history and multiple methods

Two studies examined the association between STI history and multiple methods [33, 83]. When condom use was combined with other methods or considered in the pooled result of multiple methods examined, one study found higher odds of use at last sex in adolescents 14–16 years old who had a lifetime history of STI (AOR 4.80, 95%CI; 1.80–13.10) [33]. The other also found higher odds of use, but in sexually active adolescents 15–19 years old who had an STI within the past year (AOR 8.27, 95%CI; 2.54–26.10) [83] (Table 1). Kawuki et al. [83] also reported an AOR including non-sexually active participants (AOR 38.89, 95%CI; 10.28–147.10) (Table 1). Both studies had a high risk of bias without threat to conclusion validity (Fig. 2).

#### STI history and unprotected sexual acts

Two studies examined the association between STI history and unprotected sexual acts [36, 82]. One study found that having had an STI more than three months ago was significantly associated with an increase in unprotected vaginal sex in the past three months in 18–24-year-old participants (β 5.32, 95%CI; 0.52–10.12) [36]. Another found that the odds of risky sexual behavior were higher in 15–19-year-old participants previously diagnosed with an STI (AOR 2.08, 95%CI; 1.12–3.96) [82] (Table 1). Only the study reporting on risky sexual behavior had a possible threat to conclusion validity [82] (Fig. 2).

#### STI history and condom use, according to gender

All studies that examined the effect of a history of STI on condom use only in girls (*n* = 4) inspected either dual method or multiple methods as outcomes [33, 83–85] (Tables 1 and 2). According to the results with these outcomes, all were statistically significant and associated with higher use, while none presented any threat to conclusion validity regarding risk of bias assessment [33, 83–85] (Fig. 2). No association with STI history was found regarding condom use alone in girls [84]. The study that included only boys (*n* = 1) found that adolescent boys with a history of STI had lower odds of using a condom at last sex than those without such history [81], with no threat to conclusion validity (Fig. 2). Two studies included both boys and girls. Both studies, when inspecting unprotected sex acts or risky sexual behaviors, found a positive association with STI history [36, 82]. However, no association was found regarding condom use alone [36]. Possible threats to conclusion validity remained regarding Ebuenyi et al. [82] (Fig. 2).

#### STI history and condom use, according to ethnicity or race

Three studies were conducted with African-Americans; these examined condom use alone and dual method [84], multiple methods [33], and condom use alone [81] (Tables 1 and 2). All three studies found statistically significant effects toward a positive association between history of STI and dual method or multiple methods (Table 1). One study conducted with a majority of Black non-Hispanic Americans (61%) found an increase in unprotected vaginal sex acts in boys diagnosed with an STI more than three months ago [36], with no threat to conclusion validity (Fig. 2). The one study conducted with Nigerians presented a threat to conclusion validity regarding the positive association found between STI history and risky sexual behavior (Table 1 and Fig. 2) [82]. The study conducted with Rwandans found a positive association between STI history in the past year and the use of multiple methods (Table 1), with no threat to conclusion validity [83] (Fig. 2). Finally, only one study was conducted with a majority of White Americans, which found a positive association between lifetime history of STI and dual method use in the past three months (Table 1) [85], with no threat to conclusion validity arising from bias (Fig. 2).

#### STI history and condom use, according to age

Two studies examined the association between STI history and condom use in adolescent populations with mean age above 20 years [36, 82]. One found a positive statistically significant association between STI history and unprotected vaginal sex acts [36], while the other found a statistically significant association with risky sexual behaviors [82] (Table 1). Both were assessed as having a high risk of bias, with only the one regarding risky sexual behaviors presenting a possible threat to conclusion validity (Fig. 2). The remaining studies (*n* = 5) examined the association between STI history and condom use in younger participants (≤ 18.1 mean years of age) and examined either condom use alone or dual method/multiple methods (Table 1) [33, 81, 83–85]. Those studies found the previously stated results regarding dual method/multiple methods and condom use alone, with the respective risks of bias (Fig. 2).

## Discussion

### Summary of evidence

Three types of outcomes regarding condom use were identified in only seven studies over the last decade. Most of those studies presented interpretable conclusions even in the presence of a risk of bias. Our review found that: 1) a history of STI was rarely associated with condom use alone, except when analyzed only in boys, who tended to use condoms less after having recovered from an infection; 2) younger girls with a history of STI increased their use of dual/multiple methods; 3) both boys and girls of older age reported increased unprotected sex acts after an STI; and 4) race/ethnicity cannot, at this point, be stated as influencing the association between STI history and subsequent condom use. Due to the small number of studies, the findings should be interpreted with care.

### Explanatory hypotheses supported by literature

In one of the included studies, STI history in boys was associated with a reduction in subsequent use of condom alone. Some studies showed that the association between STI history and condom use was influenced by gender. The association between a history of STI and subsequent condom use alone may disappear when the two genders are combined in the analysis. Studies that did not differentiate by gender in their analysis possibly confounded the effect. The studies that included boys did not inspect the association with the dual method or multiple methods of protection including condoms. Those two outcomes referred mostly to pregnancy prevention methods only used by girls [87]. However, studies examining contraception in adolescence have historically analyzed it in girls, who still often assume responsibility for reproductive planning and childcare [88, 89]. The lack of association or possible negative association with condom use alone could be due to adolescent boys’ taking more sexual risks than girls [90]. Boys-only analyses could show an increase in risk-taking behavior considering that tendency of higher risk-taking [90, 91]. Moreover, one of the most common STIs, chlamydia, has few complications in boys, which could influence their subsequent behaviors after infection [92]. However, the one study that found an association between STI history and condom use alone only reported it in one of the waves of their repeated cross-sectional survey, which suggests that it could not be replicated in different years [81]. This could also mean, as the authors concluded, that STI history is not a consistent predictor of condom use alone [81].

Remarkably, STI history was associated with subsequent unprotected sexual acts and risky sexual behaviors in both boys and girls, even when a history of STI appeared to increase subsequent condom use in combination with other contraceptive methods in girls. Determinants of preventive health behaviors may help explain this. In fact, those two studies combining boys and girls each had a population with a mean age over 20 years, and older adolescents are known to be more prone to adopting negative attitudes toward condom use [36]. As condom use is significantly influenced by attitudes [93], this could potentially explain the direction of the association. Studies should be replicated in older adolescents with a rigorous methodology to eliminate the risk of threat to conclusion validity that is present in one of those studies.

Girls with a history of STI increased their dual/multiple methods use. Several factors contribute to the importance of condom use in adolescent girls [74, 94]. Girls are disproportionately affected by STIs [95], and those who are infected may be warned about the serious potential impacts of STIs on their fertility [96–98]. The effects are particularly important on their reproductive health because of their anatomy, in that female fertility is vulnerable to STIs due in part to delicate vaginal mucosa [97] and possible damage and occlusion of the fallopian tubes from previous infection [98]. Moreover, these vulnerabilities can be exacerbated when girls are living in unfavorable sociocultural and economic conditions [97]. It is plausible that the clinical approach taken with girls infected by STIs may be different from that with boys. This could explain the divergence between genders with regard to the impact of STI history on subsequent condom use and extra protection regarding pregnancy.

With regard to race or ethnicity, no clear pattern appeared to influence the association between STI history and subsequent condom use. Three studies found a positive association with the dual method or multiple methods, while three others found associations with unprotected sexual acts. The only study in a White population found a positive association with dual method use. This could potentially mean that neither race nor ethnicity explain all the relationships between STI history and subsequent condom use, as gender appears to do in those seven studies. African-American girls are often disproportionately negatively affected by sexual and reproductive health conditions compared with girls from other races or ethnicities, in part because of socioeconomic factors [99]. The higher odds of infection in ethnic minorities [11, 100] could partially explain their high representation in the studies included in the systematic review. Race and ethnicity remain important in health research, notably to highlight health disparities. However, given the lack of clear consensus definitions regarding race and ethnicity in some research areas, researchers should consider carefully the definitions they apply when creating classifications for their research objectives [101]. The contexts of health determinants likely play an important role when the association of interest is observed. The sociocultural context of the individuals included in the study should also be considered.

### Limitations of the evidence

An important limitation is the small number of studies included in the review, which limited our data analysis and quantification of the effect of interest on the outcomes found. More studies could help to clarify the conclusions coming from the small number of studies, as well as to generalize findings to more populations. Most of the studies included focused on African-American adolescent girls in the USA or in African countries (Nigeria and Rwanda). The results should be interpreted with caution when applied to contexts other than those studied. These studies would need to be replicated in other contexts and with more diverse populations to enhance the external validity of the findings [102].

While STI history and subsequent condom use could be studied with longitudinal studies, cross-sectional surveys were almost always used (85.7%). Cross-sectional surveys are sources of reliable information representative of the populations under study [102]. However, longitudinal studies could be beneficial, in order to establish the important temporal sequence to causality, to exclude recall bias, and to observe changes over time [102, 103].

None of the studies included in this review differentiated between curable and incurable STIs. Future research should distinguish between different types of STIs (e.g., curable vs incurable), as they may affect preventive behaviours differently. This would help clarify the association between STI history and subsequent condom use with regard to different types of exposure.

Almost all the studies used dichotomous questions regarding condom use. A 2014 systematic review of condom use measurement called for a standardization of measures [104], as the lack of standards for condom use measurement hinders the comparability of findings across studies. According to that systematic review of 215 studies, six dimensions could be considered when condom use is the outcome of interest: partner type, temporal period, measurement scale, consistency of use, controlling for abstinence, and type of sex [104]. In particular, some studies did not limit their analysis to sexually active participants, which could confound the association between STI history and condom use. This could explain, in part, why Kawuki et al. [83] found such a high odds ratio when including all samples, which would have included low numbers of participants with STI history. While exposure timeline was variable in the studies, the temporal periods for condom use were similar in most cases. However, most of the studies did not report on consistency of use, and consistency plays an important part in the reduction of transmission during condom use [105]. It was not possible to ascertain what type of partner the condom use involved: in fact, it is known that condom use varies by partner type, being lower with a primary partner and in long-term relationships [106]. Taking into account all these elements could significantly enhance the study of the association between STI history and subsequent condom use. On the other hand, almost all the studies (*n* = 5) included socioeconomic indicators, which is sometimes an important gap in the scientific literature, even in the highest-ranked medical journal, as reported in a recent literature review [107].

The three studies conducted in a clinical setting presented a risk of selection bias. Both STI history and condom use can lead people to seek clinical consultation, which could result in a possible collider bias [102, 108]. When selecting participants in a clinical context, attention should be given to ensuring that the association of interest is free of such bias. Another important selection bias can arise when excluding participants based on missing values, especially if those values are not missing at random [109], which was done in one study without consideration of the mechanism responsible for the missing data. Multiple imputation is a useful method for handling missing values [109]. Results should be presented even when not statistically significant, which sometimes leads to selecting a high risk of bias in the 7^th^ domain of ROBINS-E.

Many other psychosocial variables could explain behavior change, such as perceived susceptibility, perceived severity, health motivation, self-efficacy, or perceived benefits or barriers [110]. However, none of the articles included in the literature review elaborated on such psychosocial variables. Future research should explore how psychosocial variables and past experiences can influence preventive actions such as condom use.

### Limitations of the review process

As mentioned earlier, articles from inception to 2012 in MEDLINE, Embase, and Web of Science were excluded, in order to focus on a ten-year generational context [54, 55, 111]. Hence, one limitation of this systematic review is that the conclusions cannot be generalized to earlier time periods. However, the results will be useful for decision-makers focused on the behaviors of contemporary youth.

### Future research

Multiple studies in this review did not consider important confounding factors, such as social and sexual networks [65, 66], other risky sexual behaviors [67, 68], education [18, 69, 70], knowledge and awareness [71–73], socioeconomic status [65, 74], healthcare resources [65, 75], and cultural and religious beliefs [65, 79]. However, those confounders are known to have a positive association with STI history and a negative association with condom use, which suggests a possible underestimation of the effect measure. Even in the presence of a high risk of bias, the conclusions about the association for many of the studies would still be the same. Furthermore, the high risk of bias concluded in all the studies also results from bias arising from the measurement of condom use. This stems from the fact that people who had STIs and those who did not could potentially have given differential information regarding condom use outcomes. However, information about condom use is a self-reported measure [104], and such a requirement could not be taken into account in ROBINS-E. All our studies were ultimately classified with a high risk of bias. It is important to note that greater attention should be given to threats to conclusion validity regarding the reasons behind the categorization in the 1^st^ and 6^th^ domains.

### Implications of research findings

The differences in the association between STI history and condom use detected between girls and boys, and between the different age groups, call for a rethinking of clinical approaches and awareness-raising interventions after an STI diagnosis. Gender-specific approaches in clinical and research settings have been beneficial in fields related to psychiatry [112], cardiovascular diseases, and osteoporosis [113]. Such approaches are, in fact, a step closer to gender equity, as they take into account the specific needs of both men and women [114]. The same can be said for age-specific interventions targeting adolescents [115]. Tailoring counseling and education practices to specific groups could minimize the risks of transmission and reinfection.

## Conclusions

Condom use in adolescents who have experienced STIs is important, as they must protect themselves from reinfections and further transmission. In line with the HBM, these results highlight factors that can influence the adoption of preventive health measures, such as condom use, among adolescents who experienced STIs. The results could help in tailoring gender-sensitive clinical approaches to STI diagnosis in adolescents. As STIs are transmitted via sexual interactions, both girls and boys should be involved in decisions on their subsequent condom use and reminded of the impact of STIs. Even in older adolescent populations, with their higher possibility of perpetuating risky sexual behaviors, STIs require serious consideration. Further studies should differentiate this issue by gender and adolescent age, examining the different potential impacts on behavior. More studies, with rigorous epidemiologic methods, should also investigate the association in young boys [88, 89]. Such results could also apply to other infectious experiences, as a curable infection could potentially modulate subsequent behaviors in adolescence.

### Supplementary Information

**Additional file 1. **Search strategies for MEDLINE (Ovid), Embase (Elsevier), and Web of Science.

## Data Availability

All data and materials used in the present systematic review are available from the corresponding author.

## Abbreviations

CI: Confidence interval
HBM: Health Belief Model
HIV: Human immunodeficiency virus
HPV: Human papillomavirus
JBI: Joanna Briggs Institute
LGV: Lymphogranuloma venereum
PECOSS: Population, Exposure, Comparison, Outcome, Study design, Study setting
PRISMA: Preferred Reporting Items for Systematic reviews and Meta-Analyses
ROBINS-E: Risk Of Bias In Non-randomized Studies – of Exposure
STD: Sexually transmitted disease
STI: Sexually transmitted infection
USA: United States of America
